# Regulation of Anticancer Styrylpyrone Biosynthesis in the Medicinal Mushroom *Inonotus obliquus* Requires Thioredoxin Mediated Transnitrosylation of S-nitrosoglutathione Reductase

**DOI:** 10.1038/srep37601

**Published:** 2016-11-21

**Authors:** Yanxia Zhao, Meihong He, Jianing Ding, Qi Xi, Gary J. Loake, Weifa Zheng

**Affiliations:** 1 Laboratory for Biotechnology of Medicinal Plants, School of Life Sciences, Jiangsu Normal University, Xuzhou 221116, China; 2 Institute of Molecular Plant Sciences, School of Biological Sciences, University of Edinburgh, Edinburgh EH9 3BF, UK; 3 Jiangsu Normal University – Edinburgh University, Centre for Biotechnology of Medicinal and Food Plants, Jiangsu Normal University, Xuzhou 221116, China

## Abstract

The medicinal macrofungus *Inonotus obliquus* widely utilized as folk medicine in Russia and Baltic countries is a source of phenylpropanoid-derived styrylpyrone polyphenols that can inhibit tumor proliferation. Insights into the regulatory machinery that controls *I. obliquus* styrylpyrone polyphenol biosynthesis will enable strategies to increase the production of these molecules. Here we show that Thioredoxin (Trx) mediated transnitrosylation of S-nitrosoglutathione reductase (GSNOR) underpins the regulation of styrylpyrone production, driven by nitric oxide (NO) synthesis triggered by *P. morii* coculture. NO accumulation results in the S-nitrosylation of PAL and 4CL required for the synthesis of precursor phenylpropanoids and styrylpyrone synthase (SPS), integral to the production of styrylpyrone, inhibiting their activities. These enzymes are targeted for denitrosylation by Trx proteins, which restore their activity. Further, this Trx S-nitrosothiol (SNO) reductase activity was potentiated following S-nitrosylation of Trx proteins at a non-catalytic cysteine (Cys) residue. Intriguingly, this process was counterbalanced by Trx denitrosylation, mediated by Trx-dependent transnitrosylation of GSNOR. Thus, unprecedented interplay between Trx and GSNOR oxidoreductases regulates the biosynthesis of styrylpyrone polyphenols in *I. obliquus*.

Phenylpropanoid-derived styrylpyrone polyphenols synthesized by the medicinal mushroom *I. obliquus* have been demonstrated to reduce the incidence of cancer, hypertension and various neurodegenerative disorders^1^. Styrylpyrone is the predominant end product of phenylpropanoid production and the precursor for the synthesis of styrylpyrone polyphenols in *Inonotus (Hymenochaetaceae*) and *Phellinus (Polyporaceae*) species^1^,^2^,^3^. Under laboratory growth conditions, however, these molecules in the culture of producing fungi are typically present at low concentrations limiting their clinical utility. Thus, understanding the regulatory machinery that controls phenylpropanoid-derived styrylpyrone polyphenols in *I. obliquus* will enable strategies to increase the production of these medicinally important molecules.

Fungal interspecific interactions enhance the transient biosynthesis of defense-related phenylpropanoid-derived natural products including styrylpyrone polyphenols. A nitrosative burst, leading to the production of the redox cue, nitric oxide (NO), is thought to be integral to this process^4^. Further, an elevated level of NO-driven S-nitrosylation, the addition of a NO moiety to a reactive protein cysteine (Cys) thiol to form a S-nitrosothiol (SNO)^5^,^6^ was also detected^7^. Although a transient NO burst results in a higher accumulation of styrylpyrone polyphenols, the yield produced in laboratory growth conditions was still significantly less than that obtained from these fungi when grown in natural habitats^8^. Thus, further insights into the regulatory machinery underpinning the synthesis of these molecules is required to scale up production of styrylpyrone polyphenols and thereby fully exploit their medicinal potential.

Phenylalanine ammonia lyase (PAL)^9^, 4-coumarate CoA ligase (4CL)^10^ and styrylpyrone synthase (SPS)^11^ are key enzymes in styrylpyrone biosynthesis. A recent study suggests that the coculture of *I. obliquus* and *Phellinus morii* promotes increased phenylpropanoid-related gene expression followed by a transient increase in the production of styrylpyrone polyphenols^4^. Further, coculture of these two fungi also triggers NO burst and the subsequent S-nitrosylation of the key enzymes in styrylpyrone biosynthesis^12^. However, mechanistic insight into how this system might be regulated remains to be established. Protein-SNO formation has emerged as a major route for the transfer of NO bioactivity. Moreover, this redox-mediated, post-translational modification has been shown to be a key regulator of protein function in eukaryotes^13^. Interestingly, the activation of phenylpropanoid metabolism in *I. obliquus* in response to biotic stress parallels that in plants, where attempted microbial infection triggers a rapid nitrosative burst of reactive nitrogen intermediates including NO, which has been shown to activate the expression of key genes integral to phenylpropanoid biosynthesis, such as *PAL*^14^.

In order to function as a biologically relevant signalling mechanism, S-nitrosylation must be transient and hence reversible^15^ and mechanisms coordinating this process have recently begun to emerge. Chief among these are the enzymes S-nitrosoglutathione reductase (GSNOR) and thioredoxin (Trx). GSNOR turns over S-nitrosoglutathione (GSNO), a mobile reservoir of NO bioactivity, formed by the reaction of NO with the antioxidant tripeptide, glutathione (GSH). GSNO can function as a natural NO donor and conversely, GSH can denitrosylate protein SNOs. Thus, NO, GSH, GSNO and S-nitrosylated proteins are in dynamic equilibrium^5^. The breakdown of GSNO by GSNOR therefore indirectly reduces total protein *S*-nitrosylation^5^. In contrast, recent findings suggest that Trx may be able to mediate direct protein denitrosylation in combination with a NADPH-dependent TRX reductase in both animals and plants^16^,^17^. Further, this SNO reductase system enables discrimination between protein-SNO substrates, providing both specificity and reversibility^16^.

In *I. obliquus,* inhibiting TrxR activity resulted in enhanced GSNOR function followed by increased formation of protein-SNOs and a reduction in accumulation of styrylpyrone polyphenols. In contrast, inhibition of GSNOR coincided with increased TrxR activity, reduced protein-SNOs and an increase in styrylpyrone polyphenols^7^. This data implies that GSNOR may curtail the denitrosylation capacity of the Trx system and consequently reduce the production of fungal styrylpyrone polyphenols following the nitrosative burst. However, the underlying mechanism remains to be uncovered. Here we show that increasing NO levels promotes total cellular denitrosylation capacity by S-nitrosylating the non-catalytic Cys40 and Cys60 residues in either *I. obliquus* thioredoxin (IoTrx) 1 or IoTrx3, respectively. Our findings suggest that GSNOR limits further increases in styrylpyrone biosynthesis by acting as a substrate for either IoTrx1 or IoTrx3 *trans*-nitrosylation, thus promoting NO removal and hence reduced activity of Trx enzymes.

## Results

### The Activity of GSNOR and TrxR Determines the Redox Status of Enzymes Involved in Styrylpyrone Biosynthesis

In order to explore the possible roles of these two oxidoredoxin proteins in regulating reversible *S*-nitrosylation of the enzymes involved in fungal styrylpyrone biosynthesis, we cocultured *I. obliquus* and *P. morii* in the presence or absence of two distinct TrxR specific inhibitors, auranofin^7^ or 1-chloro-2,4-dinitrobenzene (DNCB)^18^. In a similar fashion, we employed two diverse GSNOR specific inhibitors N6022^19^ and mithramycin A^7^ to help us clarify how the activity of GSNOR and TrxR might affect the redox status of PAL, 4CL and SPS, and their subsequent catalytic activity.

**Figure 1:**
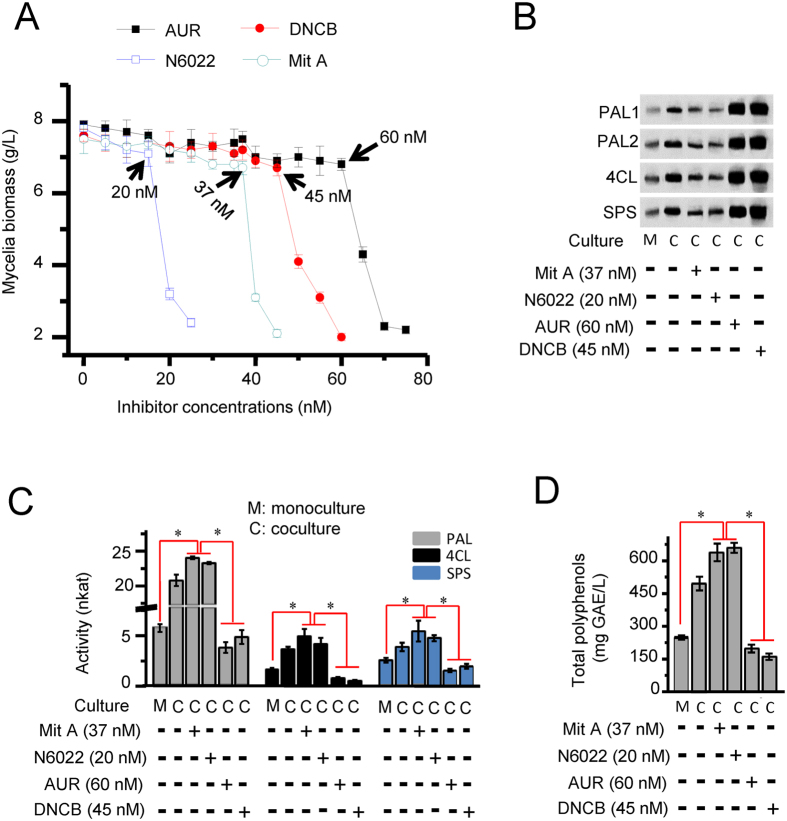
The activity of TrxR and GSNOR in *I. obliquus* affects the accumulation of styrylpyrone polyphenols, the S-nitrosylation status of the cognate enzymes and their subsequent catalytic activities. (**A**) Mycelial growth tolerance to IoTrxR inhibitors auranofin (AUR) and 1-chloro-2,4-dinitrobenzene (DNCB) and GSNOR inhibitors N6022 and mithramycin A (Mit A). The inhibitors were added simultaneously with inoculation and grown for 7 days. The data shown in each sampling point is the maximum yield of mycelial biomass determined on day 3 (n = 3 independent experiments, mean ± SD). (**B**) Catalytic activity of PAL, 4CL and SPS in the presence of TrxR or GSNOR inhibitors. (**C**) Accumulation of polyphenols by *I. obliquus* cocultured with *P. morii* in the presence of the two TrxR inhibitors AUR (60 nM) and DNCB (45 nM) or the two GSNOR inhibitors N6022 (20 nM) and mithramycin A (37 nM). (**D**) S-nitrosylation of phenylalanine ammonia lyase (PAL), 4-coumarate CoA ligase (4CL) and styrylpyrone synthase (SPS) in the presence of TrxR and GSNOR inhibitors. Coculture was conducted by inoculation of 2 ml homogenized *P. morii* into the flasks containing 200 ml four-day-old overgrown mycelia of *I. obliquus*. The mycelia were harvested on day 3 post coculture for extracting proteins. The catalytic activity of tested enzymes was demonstrated by nkat (specific activity). Polyphenols were determined by the Folin-Ciocalteu method and expressed as gallic acid equivalents (GAE), using a standard curve generated with 0–80 mg/l gallic acid. Data points from mono- and coculture represent means ± SD of three independent experiments with more than 15 samples measured at each sampling point. Asterisks indicate significant differences from the monoculture controls (*t* test, *P* < 0.05).

We first tested the suitable concentration of inhibitors. We showed that auranofin (AUR) at 60 nm, DNCB at 45 nm, N6022 at 20 nM and mithramycin A (Mit A) at 37 nM were the maximum tolerant concentrations that did not affect the accumulation of mycelial biomass (Fig. 1A). These concentrations were employed in further experiments. For combining the obligatory labeling and pulldown steps in Biotin-Switch, we employed thiopropyl sepharose beads, a thiol-reactive resin to enrich protein-SNOs (SNO resin assisted capture, SNO-RAC) to assess the formation of protein S-nitrosylation. Further, as a covalent disulfide linkage is formed between the SNO site and resin, it is more feasible for trypsinization and peptide labeling, subservient to mass spectrometric identification of SNO site^20^,^21^. The captured protein-SNOs were identified by Western Blot using specific antibodies^12^. Coculture of *I. obliquus* and *P. morii* promoted S-nitrosylation of PAL (isoforms 1 and 2), 4CL and SPS. Interestingly, addition of the GSNOR specific inhibitors, N6022 and Mit A reduced the level of S*-*nitrosylation status of these enzymes. In contrast, the TrxR specific inhibitors AUR and DNCB gave rise to increased S-nitrosylation of PAL, 4CL and SPS (Fig. 1B). Congruent with the changes of protein S-nitrosylation, the catalytic activity of PAL, 4CL and SPS (Fig. 1C) and accumulation of styrylpyrone polyphenols increased in the presence of GSNOR inhibitors and declined in the presence of TrxR inhibitors (Fig. 1D). To further validate these findings, we also knocked down the genes encoding GSNOR and IoTrx1 using vectors pCIT and pCH (Supplemental Fig. 1). Similar results were also obtained in the knockdown mutants, where knockdown of *gsnor* increased the secretion of secondary metabolites to PDA medium (Supplemental Fig. 2A) and production of total polyphenols (Supplemental Fig. 2B), reduced S-nitrosylation of PAL, 4CL and SPS (Supplemental Fig. 2C) and up-regulated enzymatic activity (Supplemental Fig. 2D). In contrast, knockdown of *IoTrx1* reduced the growth rate of *I. obliquus* on PDA medium and the production of polyphenols increased S-nitrosylation of PAL, 4CL and SPS and decreased their activity (Supplemental Fig. 2). Collectively, these data imply that the two oxidoredoxins regulate the redox status of the enzymes involved in styrylpyrone biosynthesis, with GSNOR function surprisingly promoting S-nitrosylation and TrxR reducing the level of this post-translational modification.

### Substrate Repertoire of the Trx/TrxR System and Its Denitrosylation Activity

**Figure 2:**
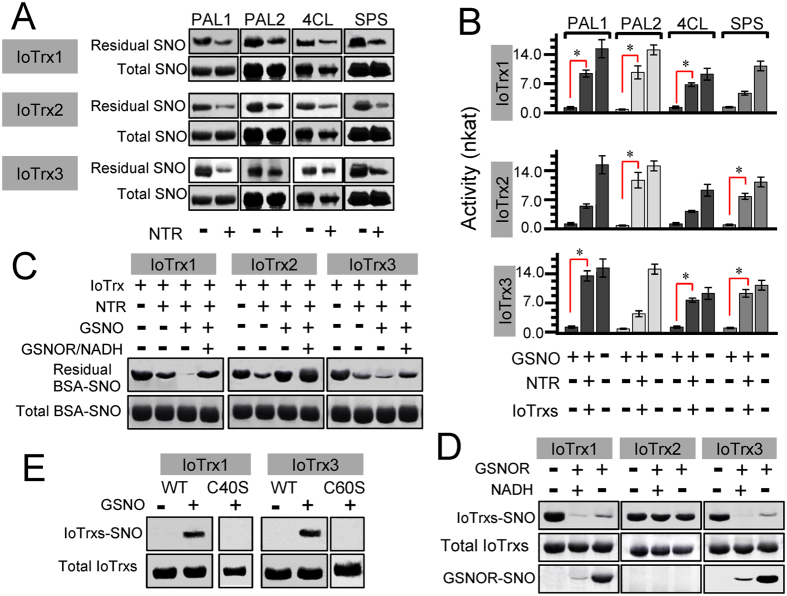
Denitrosylation activity of the three IoTrxs and their potential to reactivate the catalytic activity of the enzymes integral to styrylpyrone biosynthesis. (**A**) Denitrosylation of S-nitrosylated PAL (1 and 2), 4CL and SPS by IoTrx1, IoTrx2 and IoTrx3. (**B**) Reactivation of S-nitrosylated PAL (1 and 2), 4CL and SPS by IoTrx1, IoTrx2 and IoTrx3. The catalytic activity of tested enzymes was demonstrated by nkat (specific activity). Data points represent means ± SD of three independent experiments with more than 15 samples measured at each sampling point. Asterisks indicate significant differences from the GSNO-treated enzymes integral to styrylpyrone biosynthesis (*t* test, *P* < 0.05). (**C**) Denitrosylation capacity of GSNO-treated IoTrxs. (**D**) GSNOR inhibits the *S*-nitrosylation of IoTrx1 and IoTrx3. (**E**) Formation of IoTrxs-SNO in wild type or C40S (IoTrx1) and C60S (IoTrx3) mutants in presence or absence of GSNO. NTR, NADPH-dependent TrxR. Total, the amount of SNO (either BSA-SNO or IOTrx-SNO) before denitrosylation; Residual, the amount of SNO after denitrosylation.

To explore the potential of thioredoxin proteins (Trx) in *I. obliquus* to function as direct denitrosylases toward either PAL, 4CL or SPS, we cloned and expressed the genes based on the DNA sequences with GenBank accession numbers KM047907.1 (*TrxR*), KM047908.1 (*IoTrx1*), KR119064.1 (*IoTrx2*), KR119065.1 (*IoTrx3*) and KM047906.1 (*GSNOR*) to reconstruct oxidoredoxin systems *in vitro*, and KM519593.1 (*PAL1*), KM519594.1 (*PAL2*), KM514056.1 (*4CL*), KR069057.1 (*SPS*) to construct possible substrates. The three recombinant Trxs showed a diverse profile of activity toward these substrates. IoTrx1 and IoTrx2 exhibited high levels of denitrosylation activity toward PAL1, whereas IoTrx3 was more active against SPS (Fig. 2A). Consistent with their denitrosylation capacity, the three Trxs were also able to reactivate the catalytic activity of these enzymes (Fig. 2B). Next, we examined the denitrosylation potential of the three Trx enzymes upon GSNO treatment. Prior GSNO treatment enhanced BSA-SNO denitrosylation by IoTrx1 and IoTrx3, but impaired this activity in IoTrx2. However, in the presence of GSNOR the ability of IoTrx1 and IoTrx3 to denitrosylate BSA-SNO was reduced (Fig. 2C).

### S-nitrosylation of IoTrx1 and IoTrx3

As prior GSNO exposure subsequently enhanced the ability of both IoTrx1 and IoTrx3 to denitrosylate BSA-SNO, this natural NO donor might promote a redox-dependent post-translational modification of these enzymes. As Trx proteins have previously been reported to be S-nitrosylated^5^,^16^, we determined if the three IoTrxs could be subject to this modification. Indeed, SNO-RAC analysis^21^ suggested that either IoTrx1 or IoTrx3 could be S-nitrosylated by GSNO (Fig. 2D). As GSNOR could reduce the ability of both IoTrx1 and IoTrx3 to denitrosylate BSA-SNO, we reasoned that this might occur via the ability of this protein to remove the covalently attached NO group from IoTrx1 or IoTrx3. To test this we added GSNOR to the three IoTrxs in the presence or absence of NADH and determined the SNO status of these Trx enzymes by SNO-RAC. Surprisingly, the presence of GSNOR reversed the S-nitrosylation of both IoTrx1 and IoTrx3, but not IoTrx2. Interestingly, the absence of NADH did not result in an obvious reduction in the ability of GSNOR to remove NO from the two nitrosylated IoTrxs (Fig. 2D).

### Identification of the Site of loTrx1 and loTrx3 S-nitrosylation

**Figure 3:**
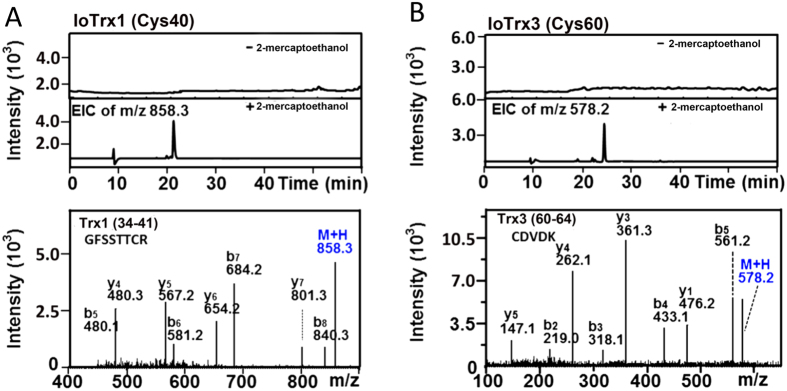
LC/MS/MS identification of the S-nitrosylation site in IoTrx1 and IoTrx3. (**A)** SNO motif in IoTrx1; (**B)** SNO motif in IoTrx3. The protein-SNOs were treated by NEM for free thiol blocking and subsequently captured by thiopropyl Sepharose beads. The captured protein-SNOs were then trypsinized followed by thorough washing to remove the tryptic peptides that were not combined with the beads. The bead binding peptides (SNO-motifs) were eluted in the absence or presence of 100 mM 2-mercaptoethanol followed by LC/MS/MS analysis. In absence of 2-mercaptoethanol the flow-though did not show any extracted ion chromatography (EIC), which implies the complete blocking of free thiols (A,B, upper panels). The 2-mercaptoethanol -eluted samples presented an EIC of m/z equal to 858.3 and 578.2, which corresponded to the expected *m/z* values for single-charged peptide SNO peptides from IoTrx1-SNO (GFSSTTCR, residues 34–41) and IoTrx3-SNO (CDVDK, residues 60–64), respectively (A,B, lower panels).

Next we sought to confirm the site of both IoTrx1 and IoTrx3 S-nitrosylation. Structurally, the two catalytic cysteines locate at different positions among all three *I. obliquus* Trxs: IoTrx1 (Cys76 and Cys79), IoTrx2 (Cys31 and Cys34) and IoTrx3 (Cys35 and Cys38). In addition, IoTrx1 and IoTrx3 harbor a non-catalytic cysteine at Cys40 and Cys60, respectively (Supplemental Fig. 3). We performed site-directed mutagenesis to change both Cys40 in IoTrx1 and Cys60 in IoTrx3 to serine to preclude SNO formation at these positions. In contrast to the wild-type proteins, both IoTrx1 Cys40Ser and IoTrx3 Cys60Ser were not S-nitrosylated following exposure to GSNO, implying Cys40 in IoTrx1 and Cys60 in IoTrx3 might be the sites of S-nitrosylation (Fig. 2E). To confirm the potential sites of IoTrx1 and IoTrx3 SNO formation, we carried out analysis by LC-MS/MS. The protein-SNOs were treated by NEM to block free thiols, which ensures the specificity of the SNO site to be captured by thiopropyl Sepharose beads after reduction. The captured protein-SNOs were then trypsinized followed by thorough washing to remove the tryptic peptides that were not combined with the beads. The bead binding peptides (SNO-motifs) were eluted with 100 mM 2-mercaptoethanol followed by LC/MS/MS analysis. In absence of 2-mercaptoethanol the flow-though did not show any extracted ion chromatography (EIC) (Fig. 3A,B, upper panel), which rules out the possibility that incomplete blocking of peptide thiols leads to significant misrepresentation as SNO-Cys-containing peptides. However, the 2-mercaptoethanol-eluted samples presented an EIC of m/z equal to 858.3 and 578.2, which corresponded to the expected *m/z* values for single-charged peptide SNO peptides from IoTrx1-SNO (GFSSTTCR, residues 34–41) and IoTrx3-SNO (CDVDK, residues 60–64) (Fig. 3A,B, lower panel), respectively. Thus, in each case the non-catalytic cysteine either Cys40 or Cys60 is the target for S-nitrosylation in IoTrx1 or IoTrx3, respectively.

### SNO Reductase Activity of IoTrx1 and IoTrx3

**Figure 4:**
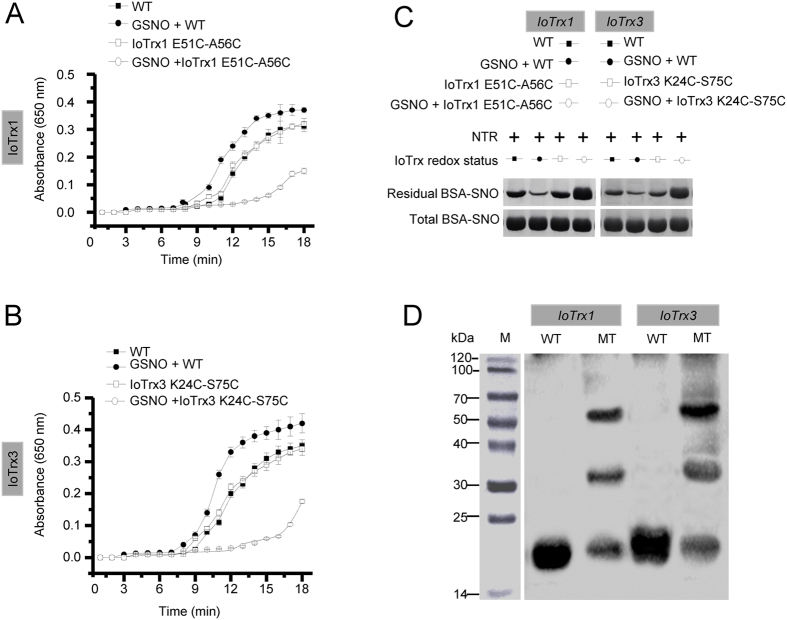
The number of non-catalytic cysteines affects the redox status of IoTrx1 and IoTrx3 and their subsequent reducing activity under nitrosative stress. (**A**) The reducing activity of IoTrx1 and its double mutant toward insulin (n = 3 independent experiments, mean ± SEM). (**B**) The reducing activity of IoTrx3 and its double mutant toward insulin (n = 3 independent experiments, mean ± SEM). (**C**) SNO-RAC analysis of denitrosylation capacity of IoTrx1 and IoTrx3 and their mutants toward BSA-SNO after treatment with GSNO. (**D**) SNO-RAC analysis of the redox status of IoTrx1 and IoTrx3 and their mutants upon treatment of GSNO. NTR, NADPH-dependent TrxR; WT, wild-type; MT, mutant. Total, the amount of SNO (BSA-SNO) before denitrosylation; Residual, the amount of BSA-SNO after denitrosylation.

Next, we determined if the presence of more cysteines in IoTrxs can further enhance their denitrosylation capacity. We reasoned that the cysteines located on the surface of Trx molecules are more likely to be combined by NO upon exposure to S-nitrosylating agents. According to the predicted 3D structures by Swiss-Model (Supplemental Fig. 4), we found that Glu51 and Ala56 in IoTrx1 and Lys24 and Ser75 in IoTrx3 are solvent exposed and prone to be S-nitrosylated once replaced by cysteines. To this end, we mutated these amino acids to conduct possible correlations between the number of cysteines and their denitrosylation capacity. The presence of two additional non-catalytic cysteines in IoTrx1 did not increase their ability to reduce either insulin or S-nitrosylated BSA relative to wild-type (Fig. 4A). Rather, IoTrx1 mutant enzyme was indistinguishable from wild-type with respect to its ability to reduce insulin (Fig. 4A) or SNO-BSA (Fig. 4C). Interestingly, upon prior GSNO treatment the reducing activity of the mutant IoTrx1 enzyme was impaired. In contrast, prior GSNO exposure enhanced insulin reducing activity in wild-type IoTrx1 (Fig. 4A). Further, SNO-RAC analysis showed that GSNO treatment of mutant IoTrx1 led to the formation of S-nitrosylated proteins with MW at 34 and 51 kDa. These SNO-proteins coincide with dimers or trimers of IoTrx1 (Fig. 4D). Similar scenario was also seen in IoTrx3 mutant, where the replacement of Lys24 and Ser75 by cysteine resulted in the reduction in reducing insulin and denitrosylase activity upon treatment of GSNO (Fig. 4B,C,D). Thus, increasing the number of non-catalytic cysteines did not affect the ability of Trx enzymes to reduce disulphide bonds within an insulin turbidity assay. Moreover, these mutants exhibited decreased denitrosylation activity toward BSA-SNO following prior exposure to GSNO. Conversely, prior GSNO treatment of wild-type IoTrx1 and IoTrx3 resulted in a striking increase in denitrosylase activity. Collectively, these data imply that increasing the number of Cys residues in Trx proteins does not enhance their ability to function as a SNO reductase.

### IoTrx1 and IoTrx3 *Trans*-nitrosylate GSNOR via A Protein-protein Interaction

**Figure 5:**
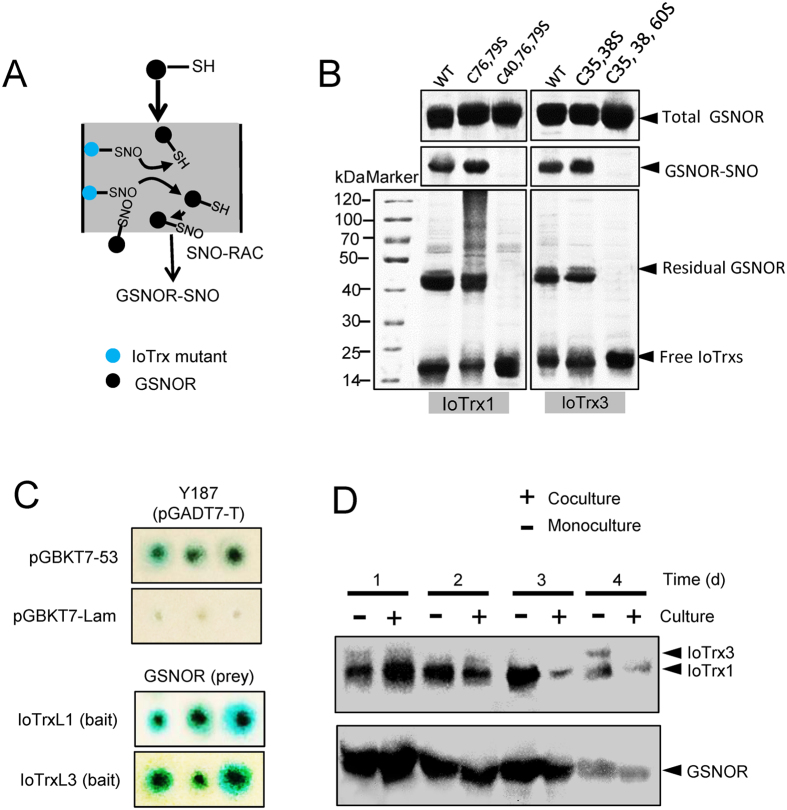
IoTrx1 and IoTrx3 *trans*-nitrosylate GSNOR by a protein-protein interaction. (**A**) Schematic of SNO-RAC, utilizing either immobilized double or triple mutants as indicated. (**B)** IoTrxs-SNO *trans*-nitrosylate GSNOR. Double mutant, C76S and C79S for IoTrx1; C35S and C38S for IoTrx3; triple mutant, C76S, C79S and C40S for IoTrx1; C35S, C38S and C60S for IoTrx3. (**C)** Yeast two-hybrid showing the interaction between GSNOR and IoTrx1 or IoTrx3 at dilution ratio of 1:500. (**D)** Coimmunoprecipitation by GSNOR monoclonal antibody or Trx antibody. The Upper lane shows immunoblotting of the proteins pulled down by GSNOR monoclonal antibody; lower lane immunoblotting of the proteins pulled down by commercialized monoclonal Trx antibody. Total, the amount of SNO (GSNOR-SNO) before denitrosylation; Residual, the amount of GSNOR-SNO after denitrosylation.

GSH can reduce a range of protein S-nitrosothiols to reconstitute the thiol group, resulting in its own conversion to GSNO, which can subsequently be turned over by GSNOR. Thus, this enzyme functions indirectly to regulate protein S-nitrosylation by controlling the level of the natural NO donor, GSNO^22^,^23^. Our results suggest that GSNOR can reverse the S*-*nitrosylation of both IoTrx1 and IoTrx3 in the absence of GSH and its enzymatic cofactor NADH. In order to further preclude the possible involvement in S-nitrosylation of catalytic residues, we mutated the two catalytic cysteines in IoTrx1 and IoTrx3. As a control, we also mutated the non-catalytic Cys residues of each Trx. To initiate these experiments, the DTT-treated GSNOR was mixed with GSNO-treated immobilized Trxs in darkness followed by sequential elution in the absence or presence of DTT (Fig. 5A). GSNOR-SNO was detected in the flow-through in the absence of DTT, which suggests that both IoTrx1 and IoTrx3 functioned as a *trans*-nitrosylase, leading to the formation of S-nitrosylated GSNOR. This was confirmed by the eluates in the presence of DTT, where the reduction of GSNOR and the presence of free Trxs were observed (Fig. 5B). To determine the S*-*nitrosylation site of GSNOR, we trypsinized the captured GSNOR-SNO and subsequently undertook LC/MS/MS analysis. The 2-mercaptoethanol-eluted samples showed an extracted ion chromatography (EIC) of m/z equal to 699.82, which coincides with the expected *m/z* values for double-charged SNO peptide (GVMPDGTSRFTCK, residues 124–136) (Supplemental Fig. 5A), and is agreement with the solvent exposed cysteine residue in the predicted Swiss-Model (Supplemental Fig. 5B). To further confirm the S-nitrosylation site in GSNOR, we also mutated Cys135 to Ser and conducted the S-nitrosylation assay. In order to avoid reduction of GSNO by GSNOR, we used CysNO as nitrosylating agent. As might be expected, mutation of Cys135 abolished the binding of NO with GSNOR (Supplemental Fig. 5C). Thus, Cys135 in GSNOR is the site for S-nitrosylation, which is identical with the solvent exposed cysteine predicted by the Swiss Model.

Protein-protein interactions can give rise to the distortion of protein interfaces and the subsequent change of protein conformation^24^, which is thought to be necessary for both *trans*-nitrosylation and S-nitrosylation/denitrosylation^5^,^19^. To further explore the molecular mechanism underpinning Trx nitrosylase function, we determined if a direct physical interaction between IoTrxs and GSNOR is required for this activity^5^ utilizing yeast two-hybrid assays. Both IoTrx1 and IoTrx3 interacted with GSNOR as indicated by the dark blue colonies which grew on the relevant selection plates (Fig. 5C). This suggests that IoTrx1 and IoTrx3 both physically interact with GSNOR in a yeast two-hybrid assay. To confirm and extend our findings, we determined if either IoTrx1 or IoTrx3 interacts with GSNOR *in vivo*. Thus, we conducted coimmunoprecipitation experiments utilizing proteins extracted from fungal mycelia over time following the initiation of a monoculture of *I. obliquus* (control) and a coculture between *I. obliquus* and *P. morii*. Addition of GSNOR antibody led to the coimmunoprecipitation of IoTrx1 at all times post coculture and IoTrx3 on day 7 in monoculture (Fig. 5D, upper panel). Further, a Trx antibody also coimmunoprecipitated GSNOR (Fig. 5D, lower panel). In addition, weaker interaction was observed between GSNOR and IoTrx1 in coculture after day 3. The interaction with IoTrx3 was not detected in coculture (Fig. 5D). Collectively, these data suggest that *I. obliquus* Trx and GSNOR enzymes interact *in vivo*.

### Interplay between IoTrxs and GSNOR Orchestrates Fungal Phenylpropanoid Metabolism

**Figure 6:**
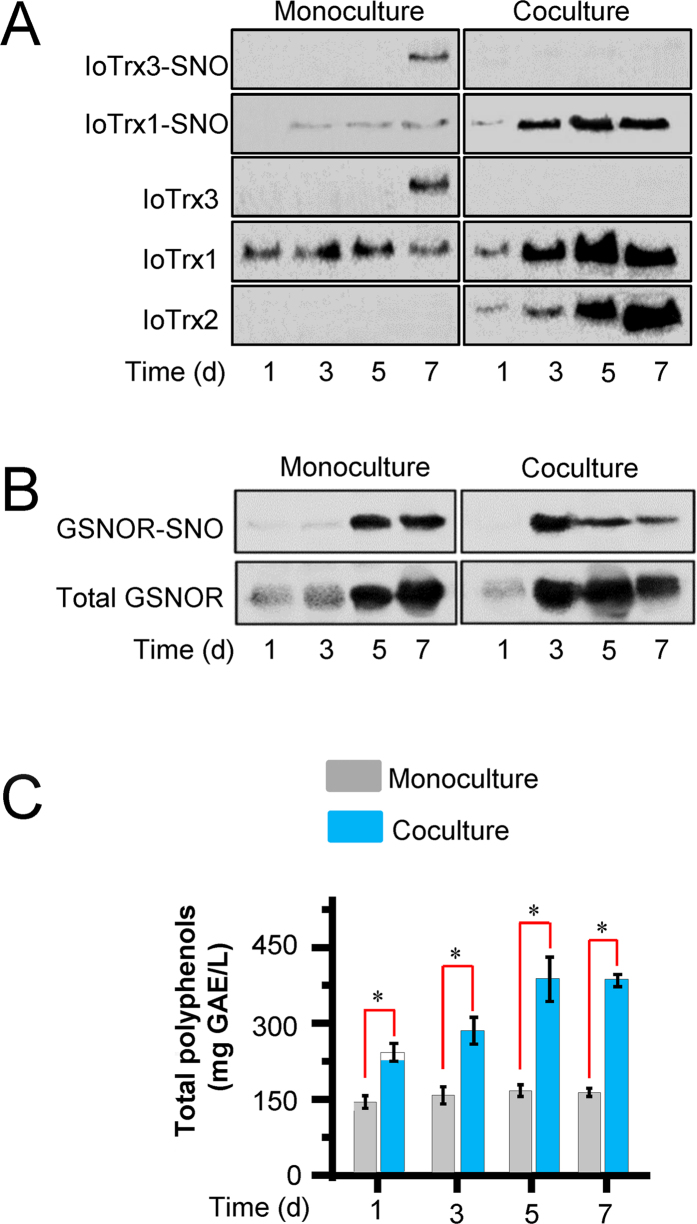
Dynamic expression and *S*-nitrosylation of IoTrxs and GSNOR in the coculture of *I. obliquus* and *P. morii* coordinates with the production of polyphenols. (**A**) Expression and *S*-nitrosylation of the three IoTrxs. (**B**) Expression and *S*-nitrosylation of GSNOR. (**C**) Production of polyphenols in coculture. IoTrxs and GSNOR were assayed using anti-C-terminal peptides of IoTrx1, IoTrx3 and GSNOR, respectively. Coculture was conducted by the inoculation of 2 ml homogenized *P. morii* into the flasks containing 200 ml four-day-old overgrown mycelia of *I. obliquus*. Polyphenols were determined by the Folin-Ciocalteu method and expressed as gallic acid equivalents (GAE), using a standard curve generated with 0–80 mg/l gallic acid. Data points from mono- and coculture represent means ± SD of three independent experiments with more than 15 samples measured at each sampling point. Asterisks indicate significant differences from the wild-type controls (*t* test, *P* < 0.05).

In order to further extend our findings, we examined dynamic expression and S-nitrosylation of Trx proteins and GSNOR in coculture of *I. obliquus* with *P. morii*. While there was a low but constant level of IoTrx1 expressed in monoculture (control), the expression of IoTrx1 displayed a time-dependent increase in coculture and reached a peak level on day 5 (Fig. 6A). In contrast, loTrx3 was not expressed during coculture. Coordinating with the increase of IoTrx1, the formation of nitrosylated IoTrx1 was also upregulated following coculture continuation (Fig. 6A). In addition, the expression of GSNOR displayed an increased pattern with culture time (Fig. 6B) with the enhanced formation of GSNOR-SNO determined on day 3 but reduced on day 5 and 7 in coculture, contrasting the increased formation on 5 and 7 in monoculture (Fig. 6B). Following the increased formation of IoTrx1-SNO and reduced formation of GSNOR-SNO in coculture, the production of polyphenols was also upregulated (Fig. 6C).

**Figure 7:**
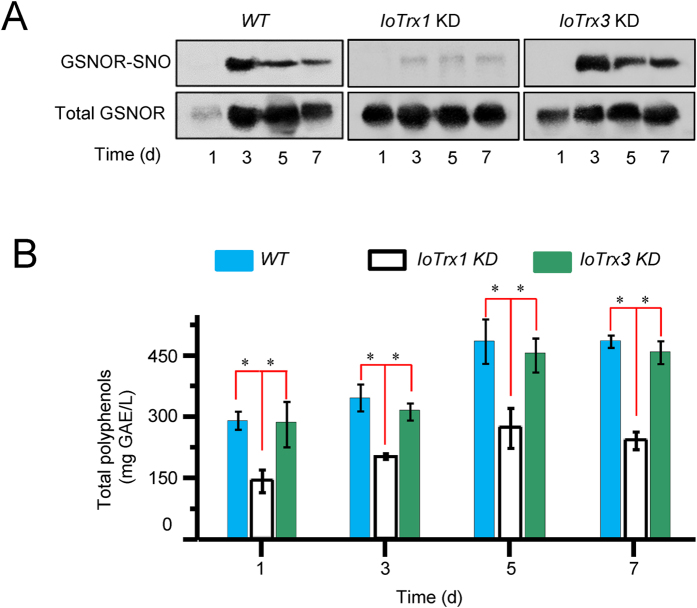
Correlation between the formation of GSNOR-SNO and production of polyphenols. (**A**) GSNOR expression and S-nitrosylation in the coculture of *wild-type (WT)* and *IoTrx1* or *IoTrx3* knockdown (KD) mutants. IoTrxs and GSNOR were assayed using anti-C-terminal peptides of IoTrx1, IoTrx3 and GSNOR, respectively. (**B**) Production of polyphenols in the cocultures of *WT* and *IoTrx1* or *IoTrx3* KD mutants. Coculture was conducted by the inoculation of 2 ml homogenized *P. morii* into the flasks containing 200 ml four-day-old overgrown mycelia of *I. obliquus*. Polyphenols were determined by the Folin-Ciocalteu method and expressed as gallic acid equivalents (GAE), using a standard curve generated with 0–80 mg/l gallic acid. Data points from wild-type, IoTrx1 and IoTrx3 represent means ± SD of three independent experiments with more than 15 mycelial samples measured at each sampling point. Asterisks indicate significant differences from the wild-type controls (*t* test, *P* < 0.05).

To identify if the function of the two potential IoTrx nitrosylases might contribute to the formation of GSNOR-SNO in coculture, we also created *IoTrx1* and *IoTrx3* knockdown mutants. Significantly, knockdown of *IoTrx1* reduced the formation of GSNOR-SNO generation (Fig. 7A) and the production of polyphenols (Fig. 7B). However, knockdown of *IoTrx3*, which is expressed only at low levels during coculture, did not affect the formation of GSNOR-SNO and polyphenols (Fig. 7).

Another notable difference in the expression profiles of Trx proteins is the presence of IoTrx2 in a time-dependent pattern in coculture (Fig. 6A). To explore if IoTrx2 might contributes to Trx/GSNOR interplay, we created a *IoTrx2* knockdown mutant. Knockdown of *IoTrx2* resulted in an increased expression of IoTrx1, IoTrx3 (Supplemental Fig. 6A) and GSNOR (Supplemental Fig. 6B), and enhanced formation of IoTrx1-SNO (Supplemental Fig. 6A), reduced formation of GSNOR-SNO (Supplemental Fig. 6B) and decreased production of polyphenols (Supplemental Fig. 6C) in coculture. In aggregate, this data implies that IoTrx2 cooperates with IoTrx1 in the reduction of protein-SNOs under nitrosative stress.

## Discussion

**Figure 8:**
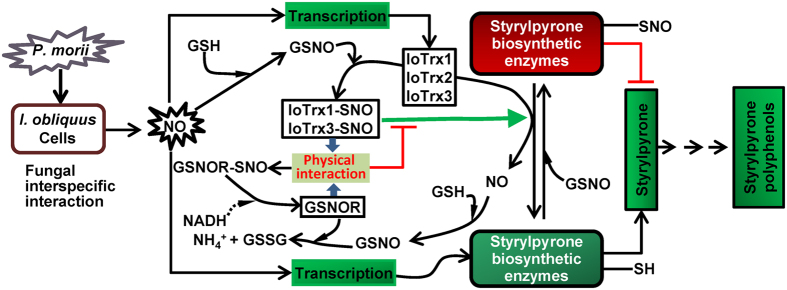
Interplay between thioredoxin proteins and GSNOR in controlling the biosynthesis of styrylpyrone during the nitrosative burst. GSH, glutathione; GSSG, glutathione in oxidized form; GSNO, S-nitrosoglutathione; GSNOR, S-nitrosoglutathione reductase; IoTrx, thioredoxin of *I. obliquus*. Black arrows indicates the regular biochemical reactions; green arrow symbolizes enhanced reaction towards denitrosylation; dashed arrow suggests the possible involvements in the biochemical reactions; green squares stand for the reaction towards biosynthesis of styrylpyrone; red lines describe the suppression of biochemical reactions; red square indicates S-nitrosylation of the enzymes integral to styrylpyrone synthesis.

Coculturing *I. obliquus* with *P. morii* triggers a burst of NO generation followed by the biosynthesis of phenylpropanoid polyphenols primarily including styrylpyrone. Inhibition of Trx function decreased the amount of styrylpyrone related polyphenols, while blunting GSNOR activity surprisingly enhanced their production. This implies a key role for these NO related oxidoreductases in the synthesis of this class of natural product. Informatively, our findings suggested that key enzymes integral to the production of styrylpyrone were targets for reversible S-nitrosylation. Inhibition of Trx function strikingly increased the extent of this redox-based, post-translational modification, decreasing enzyme activity. Conversely, inhibition of GSNOR activity reduced S-nitrosylation, increasing enzyme function. Significantly, *I. obliquus* IoTrx1, 2 and 3 all directly denitrosylated styrylpyrone synthesis-related enzymes and this SNO reductase activity was potentiated by GSNO, which resulted in the S-nitrosylation both *in vitro* and *in vivo* of IoTrx1 and IoTrx3 at their non-catalytic Cys residues, either Cys40 or Cys60, respectively. GSNO potentiation of Trx activity was abolished in the presence of GSNOR and this was independent of GSNO turnover. Interestingly, during coculture this phenomenon was found to be predominantly dependent upon IoTrx1 mediated *trans*-nitrosylation of GSNOR via physical interaction between these two oxidoreductases. Thus, Trx and GSNOR enzymes function in an intimate and cooperative fashion to regulate the production of styrylpyrone polyphenols (Fig. 8).

The phenylpropanoid pathway is integral to styrylpyrone biosynthesis and PAL and 4CL are key enzymes for the production of phenylpropanoid related metabolites^4^. The regulation of the phenylpropanoid synthesis is principally thought to occur at a transcriptional level^25^. In plants, where this pathway is well characterized, a number of transcriptional regulators of distinct classes have been shown to regulate the production of phenylpropanoids^26^,^27^. Our findings uncover a novel regulatory mechanism, S-nitrosylation, to control the activity of key enzymes integral to phenylpropanoid biosynthesis. Moreover, the activity of SPS, required for the formation of styrylpyrone from malonyl-CoA and hydroxycinnamoyl-CoA precursors^28^ was also found to be controlled by this redox modification. Thus, our data implies that post-translational control of phenylpropanoid-derived styrylpyrone biosynthesis provides another layer of regulation for the production of these metabolites.

Our findings suggest that NO appears to play a central function in the regulation of styrylpyrone polyphenol biosynthesis in *I. obliquus* during fungal interspecific interactions. A rapid nitrosative burst following fungal recognition drives NO accumulation, which activates the transcription of genes in phenylpropanoid metabolism required for styrylpyrone polyphenol biosynthesis^4^ followed by S-nitrosylation of PAL, 4CL and SPS^12^. Our data further confirm that these key enzymes in the biosynthesis of these natural products are S-nitrosylated and this modification reduces their activity, decreasing production of styrylpyrone polyphenols. Further, Trx proteins can function as denitrosylases reversing SNO formation at these enzymes, restoring their activity. Interestingly, IoTrx1 and IoTrx3 can also be S-nitrosylated at their non-catalytic Cys residues promoting their denitrosylase activity, thus providing an additional layer of regulation. Superimposed upon this is the ability of GSNOR to function as a substrate for IoTrx1 and IoTrx3 *trans*-nitrosylation, reversing SNO formation at these enzymes and decreasing their SNO reductase activity.

In Arabidopsis, addition of highly reactive NO donors like NONOate and Cys-NO to wild-type leaf extracts triggered dose-dependent suppression of GSNOR1 activity^29^. Similar inhibitory examples were also seen in GSNORs from mammalian (human) and yeast (*Saccharomyces cerevisiae*), where the binding of NO to the cysteines in GSNORs all led to their catalytic impairment^30^. In this study, we showed that the S-nitrosylated IoTrx1 is the major nitrosylase *trans*-nitrosylating the solvent exposed Cys135 in GSNOR via a physical interaction. Further, more extensive interaction between the two oxidoreductases leads to a transient increase in the formation of GSNOR-SNO, and consequently reduced denitrosylation activity of IoTrxs toward styrylpyrone synthetic enzymes, which coincides with diminished accumulation of styrylpyrone polyphenols (Fig. 6). Thus, NO mediated redox-regulation functions at multiple nodes of the regulatory machinery controlling fungal phenylpropanoid biosynthesis.

The recent discovery of protein-protein *trans*-nitrosylation reactions, the transfer of an NO group from one protein to another, has revealed a unique mechanism for targeted protein-SNO formation^31^ and conceivably could provide the basis for S-nitrosylation signaling cascades. To date, only a few examples of *trans*-nitrosylation have emerged, including between: hemoglobin and anion exchanger 1, thioredoxin and caspase-3, X-linked inhibitor of apoptosis and caspase-3, GAPDH-DHAC2 and, Cdk5 and dynamin related protein 1^17^,^32^,^33^,^34^,^35^. Our findings suggesting that IoTrx1 and IoTrx3 can *trans*-nitrosylate GSNOR expands this list beyond human proteins and further establishes these enzymes as nitrosylases.

Our data also implies a previously unrecognized level of coordination between Trx and GSNOR to regulate both the specificity and reversibility of protein S-nitrosylation in *I. obliquus*. Exploiting these unexpected findings might help guide prospective strategies toward increasing the production of medically relevant styrylpyrone polyphenols. Further, determining if this mechanism of Trx-GSNOR cooperation extends across phylogenetic kingdoms may also provide novel future targets for therapeutic and agricultural intervention.

## Materials and Methods

### Microbial Materials and Growth Conditions

The fungal strains used in this study include *Inonotus obliquus* ATCC 22881 (purchased from ATCC, USA) and *Phellinus morii* 0693 (purchased from the culture collection of Basidiomycetes of the Komarov Botanical Institute, St. Petersburg, Russia). These two fungi were maintained and cultured in the same methods detailed previously^12^. The bacteria strains used include *E. coli* DH5α and BL21 (Novagen Darmstadt, Germany). Among them, *E. coli* DH5α was used as a host for DNA cloning and *E. coli* BL21 for recombinant protein production. The two *E. coli* strains were maintained as glycol storage at −80 °C and cultured in shake flasks at 37 °C in a Luria-Bertani (LB) medium.

### Knockdown of the Genes Encoding GSNOR and IoTrx1, IoTrx2 and IoTrx3

RNA interference was used for knockdown of genes. The two complementary cDNA fragments from *GSNOR* (300 bp), *IoTrx1* (200 bp), *IoTrx* (150 bp) and *IoTrx3* (200 bp) were amplified, respectively, using the primers listed in Supplemental Table 1, and inserted to vector pCIT that flanked to the intron (Supplemental Fig. 1A,B) to form silencing construct (Supplemental Fig. 1C). The silencing construct including PtrpC and TtrpC was released by Xho1 and Sac I and subcloned to the sites of Xho1 and Sac I in vector pCH containing hygromycin resistant gene to form pCH-silence plasmids (Supplemental Fig. 1D). For preparing the competent cells, the *I. obliquus* mycelia were incubated by rotation in 10 ml osmotic medium^36^ containing lysing enzyme 45 mg (Sigma), yatalase 30 mg (Takara), glucuronidase 50 μl (Sigma), glucanase 20 mg (Sigma) and BSA 50 mg (MP Biomedicals, USA) at 37 °C for 18 h followed by protoplast capture according to the protocols described elsewhere^36^. Transformation of the plasmids were conducted on an osmotic PDA medium containing 0.1 μg/ml hygromycin B (Sigma) as the procedure previously described^36^. The positive mutants were further screened using hygromycin B containing PDA medium and used for further experiments.

### Gene Cloning, Plasmid Construction, Protein Expression and Antibody preparation

Cloning and expression of the genes involved in this study was based on the DNA sequences of the genes submitted to GenBank (https://www.ncbi.nlm.nih.gov/nuccore/?term=Inonotus+obliquus). The mycelia were withdrawn 3 days post coculture and washed three times with pure water followed by grounding in liquid nitrogen with mortar and pestle. RNA extraction, gene cloning and protein expression were performed as published previously^37^. For heterologous protein expression, the coding regions of the target genes were amplified using the primers listed in Supplemental Table 1 and inserted into vector pETMALc-H (Merck). The recombinant MBP-His-tagged proteins were purified from *Escherichia coli* BL21 (Novagen) using nickel Sepharose beads (GE Healthcare) following the manufacturer’s manual. The MBP and His tag in the recombinant proteins were cleaved by passing through a thrombin-agarose column (Sigma) according to the method described previously^38^.

For antibody preparation, the DNA sequences encoding C-terminal of PAL1 (726–826 aa), PAL2 (751–851 aa), 4CL (549–649 aa), SPS (220–295 aa), IoTrx1 (90–154 aa), IoTrx2 (70–109 aa), IoTrx3 (90–164 aa) and GSNOR (300–381 aa) were amplified according to the primers listed in Supplemental Table 1 and inserted into the vector pETMALc-H and purified from *E. coli* BL21 using the nickel Sepharose beads. The cleavage of MBP and His tags in the fusion proteins and preparation of antibodies were conducted as detailed previously^12^. The titter of anti-serum was determined as the procedure detailed elsewhere^39^. The specificity of the antiserum was assessed by blotting with total mycelial proteins. Those that resulted in a single blotting band were used for identification of tested proteins.

### Site-directed mutagenesis

Site-directed mutagenesis was carried out using the Quick-change II Site-Directed Mutagenesis Kit (Stratagen) according to the manufacturer’s instructions. The point-mutated plasmid pETMALc-H-Xpm (where Xpm is the target gene for point-mutation) was constructed by PCR using the template plasmid pETMALc-H-X (X is the gene targeted for mutation) and the primers listed in Supplemental Table 1. The constructed plasmid pETMALc-H-Xpm was transformed into *E. coli* BL21 for expression followed by thrombin cleavage and purification by MBP Sepharose High Performance column (5 ml) (BioVision).

### S-nitrosylation and Denitrosylation

Protein S-nitrosylation and denitrosylation were assayed using S-nitrosothiol resin assisted capture (SNO-RAC)^20^. For determining the changes of protein S-nitrosylation and denitrosylation in mycelia, the mycelial samples were grounded in liquid nitrogen at darkness by mortar and pestle followed by protein extraction using HEPES buffer (11.95 g HEPES, 7.42 g NaCl, 100 ml glycerol, 1 mM phenylmetanesulfonylfluride (PMSF, Sigma), 1 μg/ml pepstanin and leupeptin, pure water up to 1 L, pH 7.4) at room temperature and concentration using 0.5 ml Ultra centrifugal filters (3 kDa cutoff) (Millipore). For assaying S-nitrosylation of recombinant PAL, 4CL, SPS and three IoTrxs, the protein samples were S-nitrosylated with 0.5 mM GSNO (Sigma) for 20 min in darkness at room temperature. For purification of all treated samples, otherwise stated specifically, the excessive nitrosylating agents (GSNO or Cys-NO) or reductant (DTT) were removed by repeated filtration using Ultra-0.5 ml centrifugal filters (3 kDa cutoff). The free thiols in the protein samples (mycelial and GSNO-treated recombinant proteins) were blocked by N-ethylmaleimide (NEM) (Sigma) followed by precipitation with three volumes of acetone at −20 °C for 20 min to remove the excessive NEM as detailed previously^40^. The precipitated protein was centrifuged at 5000 g for 5 min and the pellet was washed extensively with three volumes of 70% acetone and resuspended in HENS buffer (100 mM HEPES, 1 mM EDTA, 0.1 mM neocuproine and 1% DSD, Ph 7.7). The resuspended samples were then loaded to 80 μl thiopropyl Sepharose beads (GE Healthcare) in the presence of 40 mM sodium ascorbate and rotated in the dark for 1.5 h at room temperature and then overnight at 4 °C followed by washing four times according the procedure described previously^18^. The captured proteins were eluted with 30 μl HENS/10 buffer (HENS diluted 1:10) containing 100 mM 2-mercaptoethanol (Sigma) at room temperature for western blot analysis. For S-nitrosylation site assay of IoTrxs, the captured proteins were also trypsinized with the same buffer containing 2 μg/ml trypsin (YaxinBio, Shanghai, China) at 37 °C for 18–24 h in darkness. Digestion was terminated by addition of 0.5 mM PMSF. For assaying S-nitrosylation site in GSNOR, we treated GSNOR with 500 μM Cys-NO (prepared according to the protocols described previously^41^) for 20 min in darkness instead of GSNO so as to preclude enzymatic reduction of GSNO by GSNOR. After removal of the excessive Cys-NO by filtration, GSNOR-SNO was captured using thiopropyl Sepharose beads followed by trypsin digestion and elution of resin-captured peptides from IoTrxs or GSNOR in the absence or presence of 100 mM 2-mercaptoethanol. The flow-throughs were subject to LC-MS/MS for identification of S-nitrosylation site according to the procedures described previously^40^.

Denitrosylation was assayed by the reduction in the amount of protein-SNO in 1 ml HENS buffer containing 100 nM IoTrxs, 100 nM TrxR and 450 μM NADPH. Prior denitrosylation assay, the recombinant proteins including IoTrxs, TrxR and GSNOR were separately treated with 50 mM DTT for 20 min at room temperature. In parallel, BSA and the recombinant proteins PAL1, PAL2, 4CL and SPS were treated by 0.5 mM GSNO to prepare S-nitrosylation substrates at room temperature in darkness for 30 min. After purification, 1 mg protein-SNO substrates was added into IoTrx/TrxR/NADPH system to initiate the reaction at room temperature for 30 min. The remaining protein-SNO was determined by SNO-RAC and western blot using antibodies anti-BSA, A1133 (Thermo Fisher), anti-PAL1, PAL2, 4CL and SPS (self-prepared with major specific blotting bands against antigens)^12^.

For evaluating the denitrosylation activity of IoTrxs in the presence of more cysteines, we replaced the coding sequences of Glu51 and Ala56 in IoTrx1 and Lys24 and Ser75 in IoTrx3 by cysteines according to the predicted 3D structures of Swiss-Model by commercial synthesis (Springen, Nanjing, China) and inserted the mutated DNA sequences into the vector pET28a (+). The purified mutant proteins (10 μM), either treated with 200 mM DTT or 0.5 mM GSNO were subject to assessing denitrosylation activity to BSA-SNO by SNO-RAC, and the reducing activity to insulin by incubating with freshly prepared bovine insulin (1.25 mg/ml) at 25 °C for turbidity measurement at 650 nm every 1 min^42^.

### LC-MS/MS Data Analysis

Desalted tryptic peptides were analyzed on an 1100 series nLC coupled to an XCT plus ion trap mass spectrometer (Agilent) using an 11-cm fused silica capillary column (100 μm i.d.) packed with MonitorC-18 (5 μm) (Column Engineering, CA) with the flow rate at 700 nl/min according to the protocols described previously^43^ with minor modifications. Briefly, we used the mobile phase consisting of 0.1% formic acid HPLC grade water (A) and acetonitrile (B) to elute the peptides in the following gradient elution program: 0–3 min 99% A, 3–5 min 95%, 5–30 min, from 95% A to 72% A, then to 20% A at 35 min and held to 40 min. MS/MS spectra were acquired using a full scan followed by data-dependent scans on the four most intense precursor ions. Precursors that were detected twice within 15 s were put on a dynamic exclusion list for a period of 60 s. Analysis of MS/MS spectra for peptide identification was conducted by protein database searching with SPECTRUM MILL software (Agilent Technologies). Raw MS/MS spectra were initially processed to retrieve MS/MS spectra that could be attributed to at least two y- or b-series ions, and only those spectra were searched against the SwissProt fungal database. Key search parameters were minimum matched 50% peak intensity. The threshold for peptide identification was a SPECTRUM MILL score of >10% and SPI% (the proportion of assigned spectrum intensity of total spectrum intensity) of >70%^40^. All MS/MS spectra were confirmed by manual inspection.

### Mechanistic Analysis of the Removal of NO from Nitrosylated IoTrxs by GSNOR

The catalytic cysteines in DNA sequences of *IoTrx1* (KM047908.1) and *IoTrx3* (KR119064.1) were point-mutated with QuikChange II Site-Directed Mutagenesis Kit (Stratagen) (using the primers listed in Supplemental Table 1) and fused via their C-terminals with streptavidin binding peptide and inserted in vector pET28a (+) (Merck). The recombinant mutant proteins were purified from *E. coli* BL21 using nickel Sepharose beads (BioVision). The purified IoTrx mutants (50 μg) were immobilized by 100 μg streptavidin Sepharose beads in the presence of 20 mM DTT for 1 h at 4 °C and subsequently washed to remove the reductant^18^. After thorough washing the immobilized IoTrxs were treated with 0.5 mM GSNO in darkness at room temperature for 20 min followed by elimination of the excessive GSNO with 0.5 ml-Ultra centrifugal filters (3 kDa cutoff). To initiate the experiments, the purified DTT-treated recombinant GSNOR was mixed with the immobilized double mutant of IoTrx1 (C76S, C79S) and IoTrx3 (C35S, C38S) or triple mutants in darkness at 4 °C overnight followed by elution with TBS (pH 7.5). The flow-through was subject to SNO-RAC analysis using GSNOR or Trx antibodies. The immobilized IoTrxs and residual GSNOR were eluted with TBS (pH 7.5) containing 100 mM DTT for 30 min at room temperature and assayed by Western Blot using self prepared anti-GSNOR and anti IoTrx antibodies^12^.

### Co-immunoprecipitation Assay

Mycelial proteins were extracted with HEPES buffer containing 50 mM HEPES (pH 7.4), 137 mM NaCl, 10% glycerol, 1 mM PMSF, 1μg/ml pepstanin and leupeptin by rotating at 4 °C for 30 min followed by centrifugation at 12,000 g for 15 min at 4 °C. After centrifugation, the supernatant was subject to preclearing and antibody precipitation for SDS-PAGE analysis as described previously^44^.

### Yeast Two Hybrid Assays

Yeast two hybrid assays were conducted using Matchmaker^TM^ Gold Yeast Hybrid System (Clontech) according to the users’ manual. Briefly, the coding regions of *IoTrx1* and *IoTrx3* were inserted into the vector pGBKT7 to construct bait plasmids that were transformed to Y2HGold yeast strain. The DNA sequence coding for GSNOR (KM047906.1) were cloned to the vector pGADT7 to construct prey plasmids that were transformed to yeast Y181 strain. Yeast two hybrid assays were initiated by the mating of bait and prey transformants and the diploid yeast cells were then plated on the medium containing X- α- Gal and aureobasidin A (DDO/X/A). Y2HGold transformants of pGBKT7–53 and pGBKT7-Lam mating with pGADT7-T y187 transformant were used as positive and negative controls, respectively.

### Enzymatic Assays

Mycelial or recombinant proteins were used for enzymatic assay. PAL activity was determined as described^45^. 4CL activity was conducted as described previously^10^. SPS assay was performed by HPLC based on the protocols published elsewhere^28^. For assaying the activity of recombinant proteins, the tested enzymes were treated by 20 mM DTT to reduce the possible protein-SNOs during extraction and purification. The excessive DTT was removed by filtration prior enzymatic assay.

### Statistical analysis

All the measurement results are statistically analyzed using SPSS v.22 (IBM, USA). Data points represent means ± SD of three independent experiments with more than 15 samples measured at each sampling point. The difference between the experimental and control groups is perceived as statistically significant when P values are less than 0.05 in *t* test.

## Additional Information

**How to cite this article**: Zhao, Y. *et al.* Regulation of Anticancer Styrylpyrone Biosynthesis in the Medicinal Mushroom *Inonotus obliquus* Requires Thioredoxin Mediated-transnitrosylation of S-nitrosoglutathione Reductase. *Sci. Rep.* **6**, 37601; doi: 10.1038/srep37601 (2016).

**Publisher’s note**: Springer Nature remains neutral with regard to jurisdictional claims in published maps and institutional affiliations.

## Supplementary Material

Supplementary Information
