# Supplementary material for: Regulation of Anticancer Styrylpyrone Biosynthesis in the Medicinal Mushroom Inonotus obliquus Requires Thioredoxin Mediated Transnitrosylation of S-nitrosoglutathione Reductase
Source: Sci Rep. 2016 Nov 21;6:37601. doi: 10.1038/srep37601 (PMC5116637; doi:10.1038/srep37601)
Supplement: Supplementary Information [file srep37601-s1.pdf]

Regulation of Anticancer Styrylpyrone Biosynthesis in the Medicinal Mushroom Inonotus obliquus Requires Thioredoxin Mediated Transnitrosylation of S-nitrosoglutathione Reductase

Supplementary Information

Yanxia Zhao[1], Meihong He[1], Jianing Ding[1], Qi Xi[1], Gary J Loake[2,3], Weifa Zheng1*

[1]Laboratory for Biotechnology of Medicinal Plants, School of Life Sciences, Jiangsu Normal University, Xuzhou 221116, China
[2]Institute of Molecular Plant Sciences, School of Biological Sciences, University of Edinburgh, Edinburgh EH9 3BF, UK
[3]Jiangsu Normal University – Edinburgh University, Centre for Biotechnology of Medicinal and Food Plants, Jiangsu Normal University, Xuzhou 221116, China

* **Address for correspondence:**

Weifa Zheng Ph. D & Professor

Laboratory for Biotechnology of Medicinal Plants, School of Life Sciences, Jiangsu Normal University, Xuzhou 221116, China
Tel:86-516-83403179
Email: yyzw@jsnu.edu.cn

Figure S1

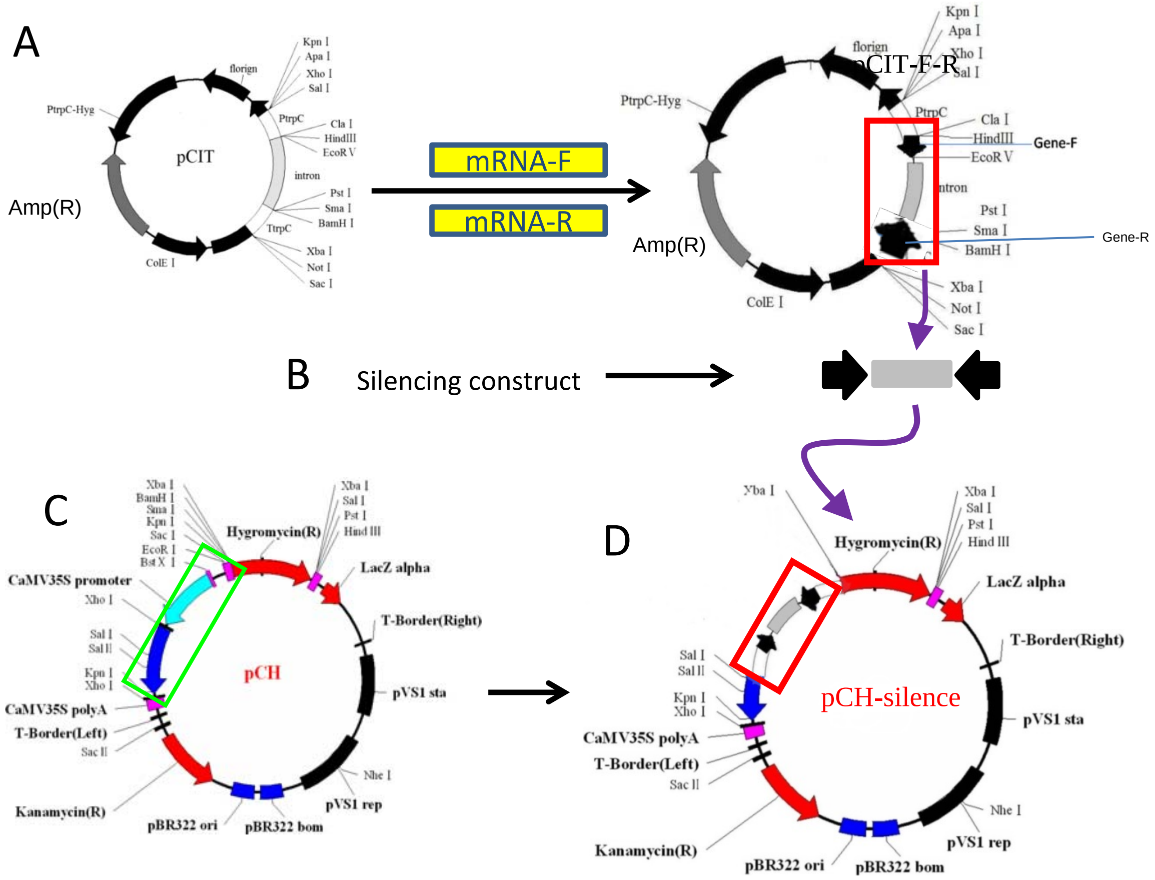

Fig. S1 Strategies of gene knockdown by RNA interference. (**A**) vector pCIT containing an intron flanked by MCS; (**B**) The mRNA fragment of target gene was fused with the two ends of intron complementarily to form silencing construct. (**C**) The vector pCH containing hygromycin resistant gene were linearized by Xho I and Sac I. (**D**) The silencing construct was inserted to vector pCH to construct pCH-GSNOR or pCG-IoTrx1,2,3 knockdown plasmid.

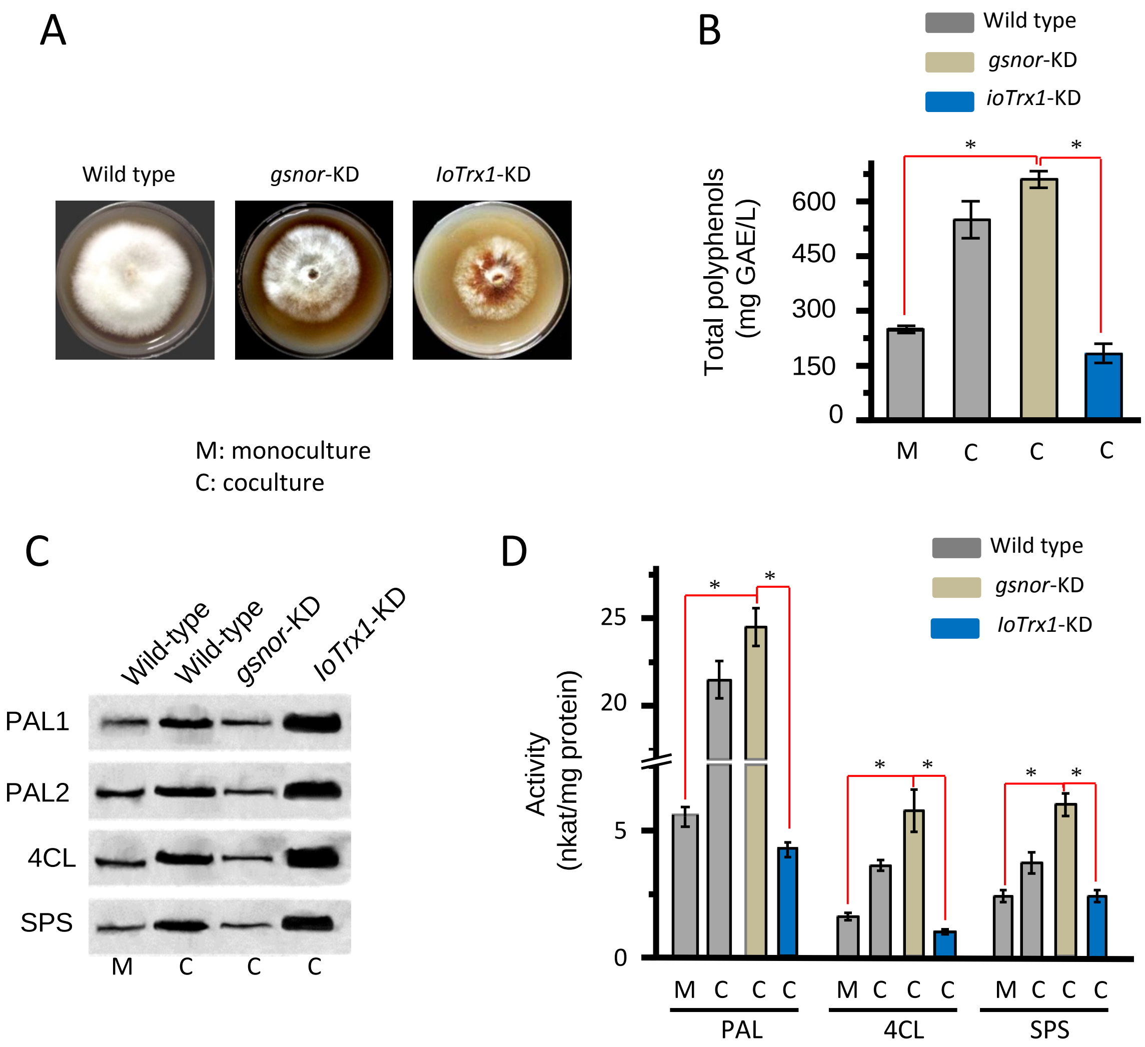

Fig. S2 Knockdown of the genes encoding GSNOR and IoTrx1 affects *S*-nitrosylation of the enzymes integral to styrylpyrone biosynthesis, their subsequent catalytic activities and accumulation of styrylpyrone polyphenols. (**A**) Phenotype of *gsnor*- and *IoTrx1*-knockdown strain (*gsnor*-KD, *ioTrx1*-KD). (**B**) Accumulation of polyphenols by *I. obliquus* wild-type or knockdown mutants cocultured with *P. morii*; (**C**) *S*-nitrosylation of phenylalanine ammonia lyase (PAL), 4-coumarate CoA ligase (4CL) and styrylpyrone synthase (SPS) in in wild-type or knockdown mutants cocultured with *P. morii*. (**D**) Catalytic activity of PAL, 4CL and SPS in wild-type or gsnor-knockdown mutant cocultured with *P. morii.* The catalytic activity of tested enzymes was demonstrated by nkat (specific activity). Polyphenols were determined by the Folin-Ciocalteu method and expressed as gallic acid equivalents (GAE), using a standard curve generated with 0-80 mg/l gallic acid. Data points from mono- and coculture represent means ± SD of three independent experiments with more than 15 samples measured at each sampling point. Asterisks indicate significant differences from the wild-type controls (*t* test, *P*< 0.01).

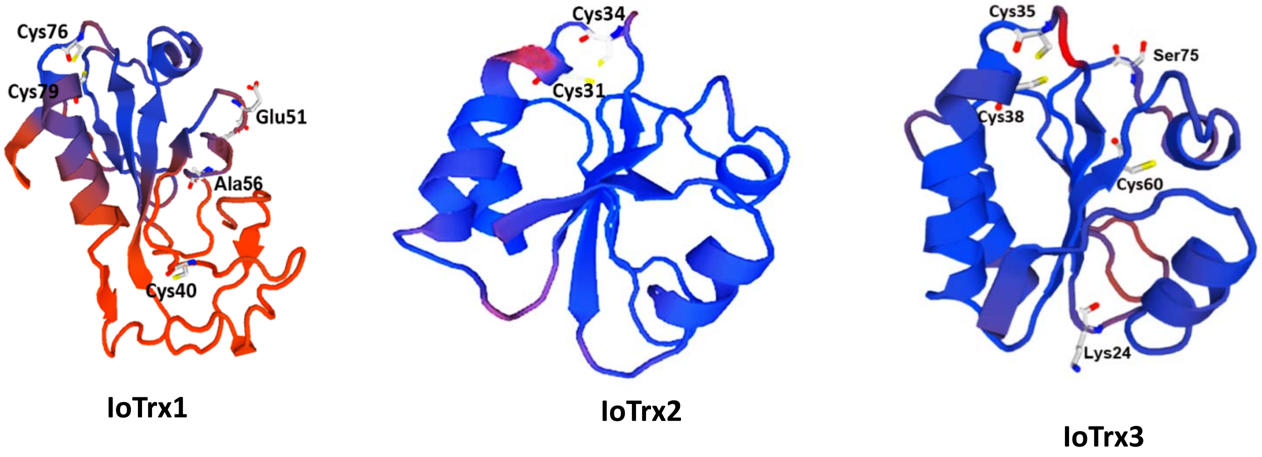

Fig. S3 Predicted structures of IoTrx1, IoTrx2, IoTrx3 and GSNOR predicted by Swiss-model.

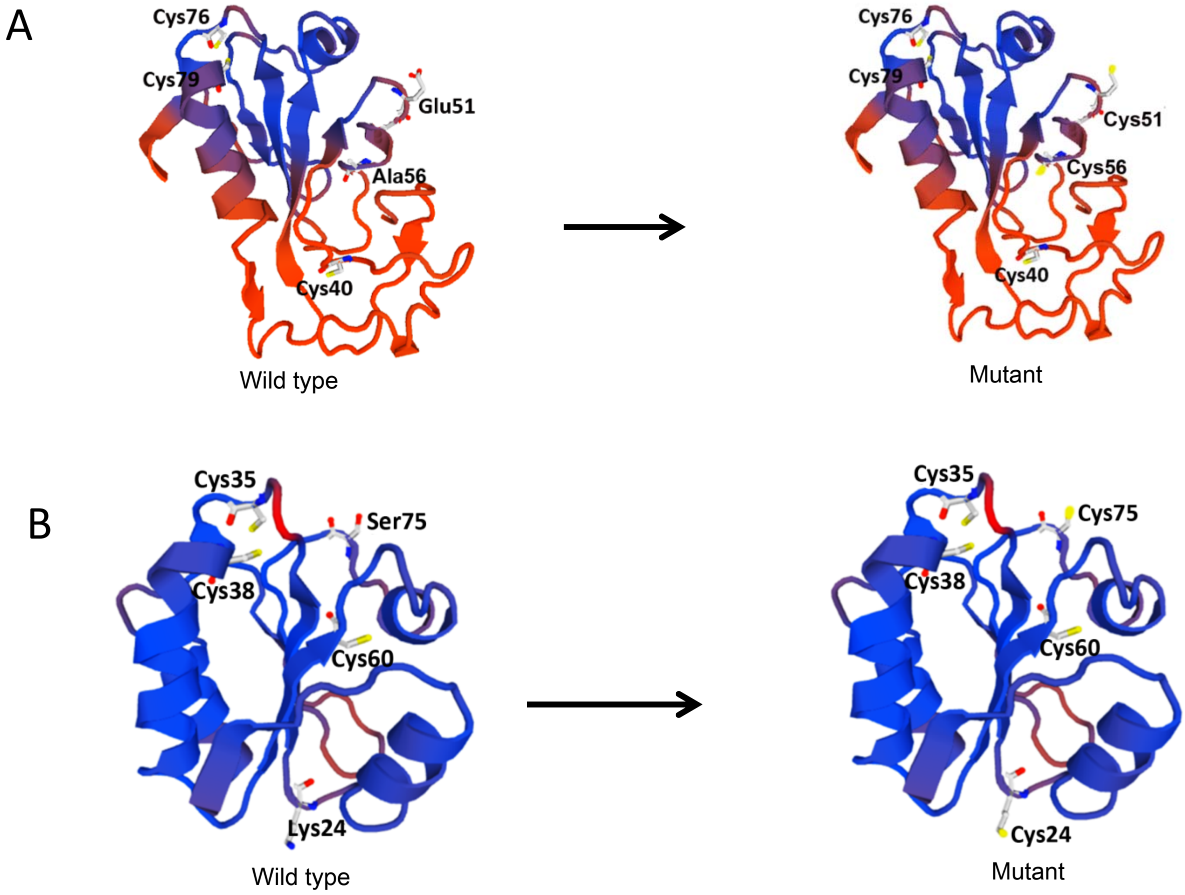

Fig. S4 Strategies for replacement by cysteines in IoTrx1 and IoTrx3 according to the structures predicted 3D Swiss-modeling. (**A**) IoTrx1. (**B**) IoTrx3.

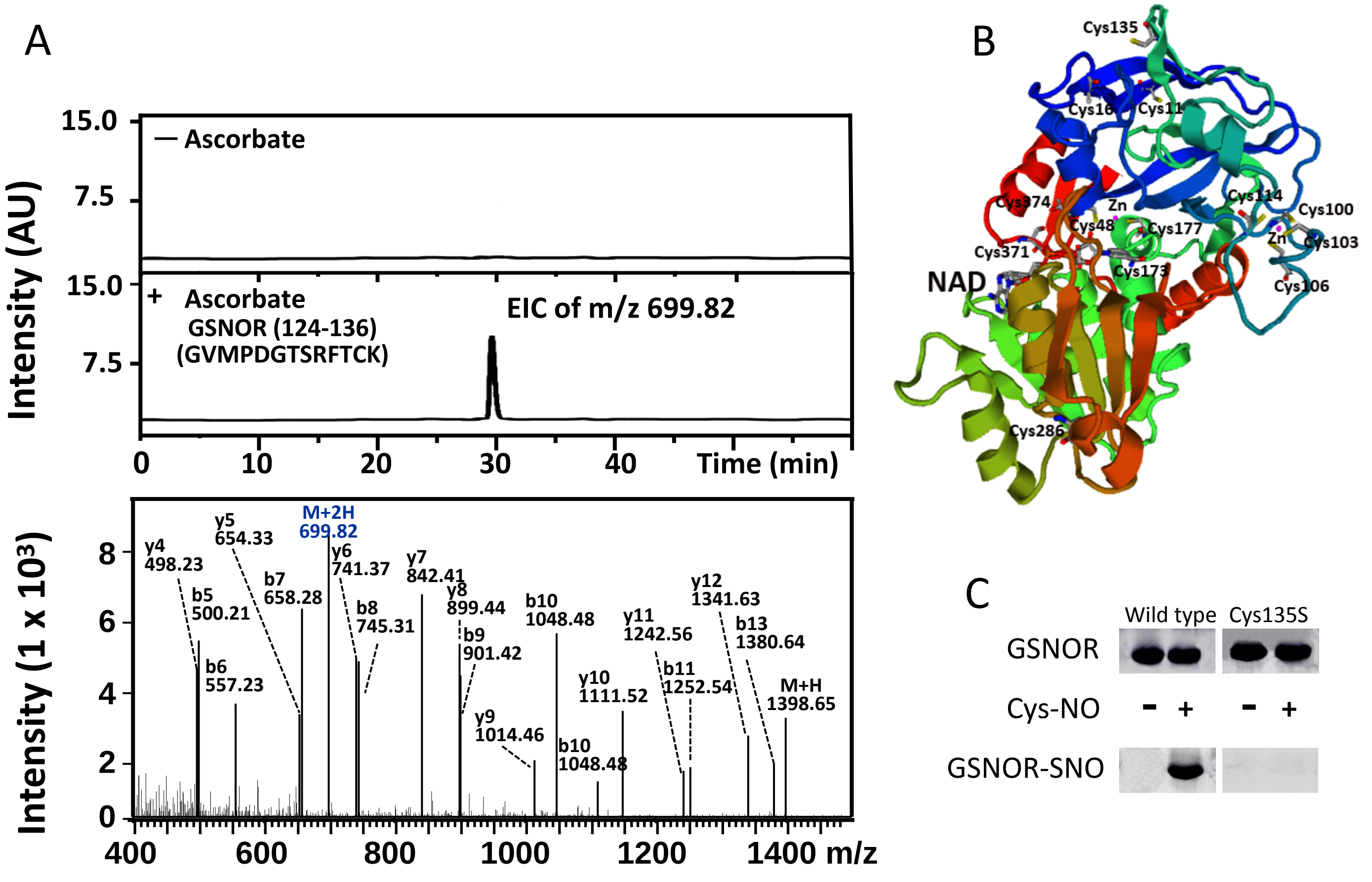

Fig. S5 Analysis of SNO motif in GSNOR**.** (**A**) LC/MS/MS identification of the *S*-nitrosylation motif in GSNOR. A single chromatographic peak was detected in each of the EIC at m/z 699.82, which corresponds to the expected m/z values from double-charged SNO peptide GVMPDGTSRFTCK. (**B**) Predicted 3D structure of GSNOR by Swiss-Model. (**C**) S-nitrosylation of wild-type and Cys135Ser mutant GSNOR by Cys-NO. GSNOR was S-nitrosylated using 500 μM of Cys-NO for 20 min in darkness. Excessive Cys-NO was removed by filtration using 3 kDa cutoff filters.

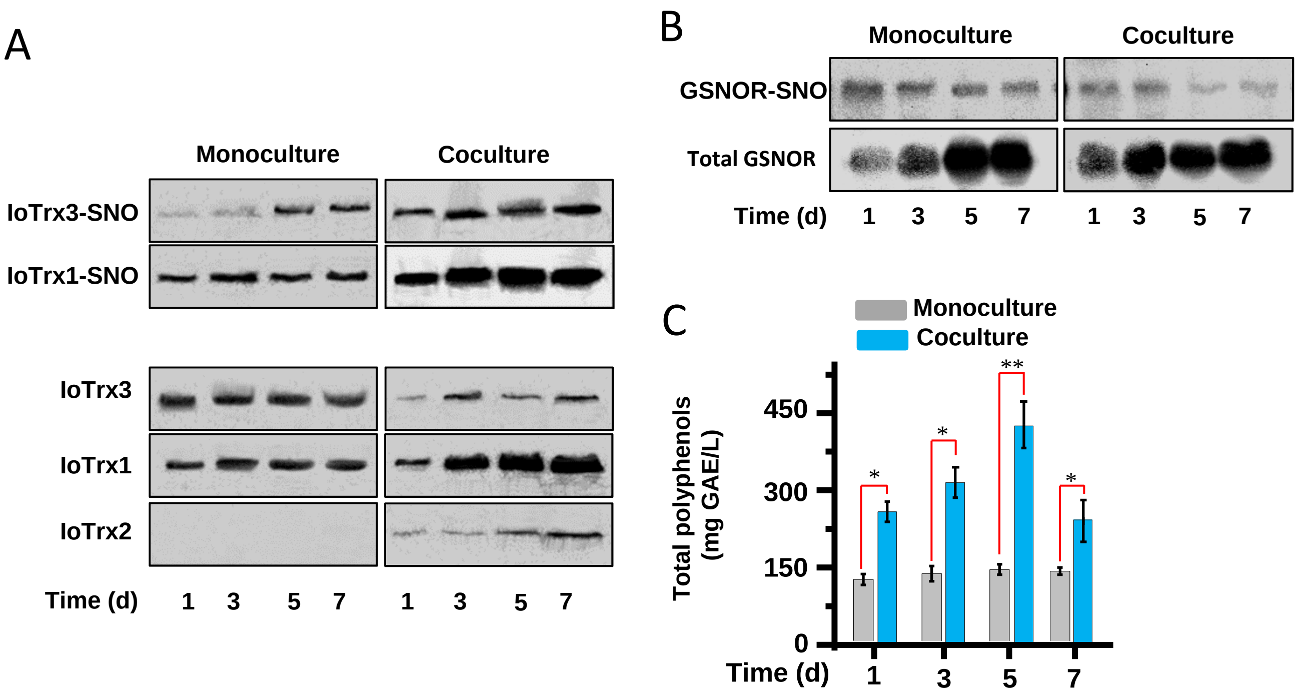

Fig. S6 Dynamitic expression and S-nitrosylation of IoTrxs and GSNOR in *IoTrx2* Knockdown mutant and their coordinating production of polyphenols in coculture of *I. obliquus* with *Phellinus morii*. (**A**) Expression and S-nitrosylation of the three IoTrxs. (**B**) Expression and the S-nitrosylation of GSNOR. (**C**) Production of polyphenols. Monoculture: a total of 20 ml *I. obliquus* mycelia homogenate was inoculated into 200 ml culture medium for submerged culture at 26^{0}C; Coculture: a total of 2 ml homogenized *P. morii* was inoculated into the flasks containing 200 ml four-day-old overgrown mycelia of *I. obliquus*. Polyphenols were determined by the Folin-Ciocalteu method and expressed as gallic acid equivalents (GAE), using a standard curve generated with 0-80 mg/l gallic acid . Data points from mono- and coculture represent means ± SD of three independent experiments with more than 15 samples measured at each sampling point. Asterisks indicate significant differences from the wild-type controls (*t* test, *P< 0.05, **P<0.01).

Table S1 Oligonucleotides used in the study

Primer name	Oligonucleotide sequences	Experiments
RPAL-1F	AGCCCATGGGTCGCTGGATCCTAAGACGTTTGAGATTGTTG	Antibody prep
RPAL-1R	TGGTGCTCGAGTGCGGCCGCTCACGCTATCCATTCTCC	
PAL_2 F	AGCCCATGGGTCGCTGGATCCAGGAGATTGCGAAAGCGGTG	Antibody prep
PAL_2 R	TGGTGCTCGAGTGCGGCCGCTTTCCCACCGTCCAGTCATTC	
4-CL- F	AGCCCATGGGTCGCTGGATCCGAATCAAGAAGGCGAAGTTC	
4-CL- R	TGGTGCTCGAGTGCGGCCGCTGCACCCATGCAAAGAGATA	Antibody prep
sps-1 F	AGCCCATGGGTCGCTGGATCCATGGTTGCTGCTCCAGGTT	Antibody prep
sps-1R	TGGTGCTCGAGTGCGGCCGCGAGTGGAAAACGGGGTA	
IoTrx1 F	AGCCCATGGGTCGCTGGATCCGAATCCGTCAAGACGGGAAGC	Antibody prep
IoTrx1 R	TGGTGCTCGAGTGCGGCCGCTTAGACTCGGTGGCCATCTTGCGT	
IoTrx2 F	AGCCCATGGGTCGCTGGATCCCAGGGCGATGCCTACGTTCG	Antibody prep
IoTrx2 R	TGGTGCTCGAGTGCGGCCGCTTATTGGTGCCGTAACTCACAC	
IoTrx3 F	AGCCCATGGGTCGCTGGATCCAACCGCAATTTGTGCGGGGTG	Antibody prep
IoTrx3 R	TGGTGCTCGAGTGCGGCCGCTTACACCCAATCTCCTCCCGT	
IoTrx1F-1	CCATCGATTGGACCATGCAAGATGATG	Gene knockdown
Iotrx1R-1	CCCAAGCTTTTATCCTTTTGCATCGCACC	
IoTrx1 F-2	CGCGGATCCTGGACCATGCAAGATGATG	Gene knockdown
IoTrx1 R-2	TCCCCCGGGTTATCCTTTTGCATCGCACC	
IoTrx2 F-1	CCATCGATTATGACGGTCACCGTAGTCAAG	Gene knockdown
Iotrx2 R-1	CCCAAGCTTTACACCCAATCTCCTCCCGT	
IoTrx2 F-2	CGCGGATCCTATGACGGTCACCGTAGTCAAG	Gene knockdown
IoTrx2 R-2	TCCCCCGGGTTTACACCCAATCTCCTCCCGT	
IoTrx3 F-1	CCATCGATTATGGCCAATAACTTAAACGAAATATCC	Gene knockdown
Iotrx3 R-1	CCCAAGCTTCAGGTTGATAATTCCACCCG	
IoTrx3 F-2	CGCGGATCCTATGGCCAATAACTTAAACGAAATATCC	Gene knockdown
IoTrx3 R-2	TCCCCCGGGTTCAGGTTGATAATTCCACCCG	
GSNOR F-1	CCATCGATAAGTGCAAGGCTGCAGTATG	Gene knockdown
GSNOR R-1	CCCAAGCTTCCCTTCCCGCATAAGTTCGTC	
GSNOR F-2	CGCGGATCC AAGTGCAAGGCTGCAGTATG	Gene knockdown
GSNOR R-2	TCCCCCGGG CCTTCCCGCATAAGTTCGTC	
pm4CLC399S F	TACATTCTATCACGTCCGGGGCGGCTCCTCTTGGTTCGGA	Point-mutation and SNO
pm4CLC399S R	GAGGAGCCGCCCCGGACGTGATAGAATGTAAGGAGCTGAA	motif identification
pmSPSC142S F	ATTCCAAGAACGCATCTTACGGCTCGACAGCTGCATTGTT	Point-mutation and SNO
pmSPSC142S R	GCTGTCGAGCCGTAAGATGCGTTCTTGGAATCGATTCCTT	motif identification
pmPAL1 F	TAGAGCAAGCAGCTTCCCTTAGGGACTTTATCACTTCCTT	Point-mutation and SNO
pmPAL1 R	TGATAAAGTCCCTAAGGGAAGCTGCTTGCTCTAGATTCGC	motif identification
pmPAL2 F	CTACTAGCCCAGGCAGCCTGCCTCAAGGACTTCATTGCTT	Point-mutation and SNO
pmPAL2 R	AATGAAGTCCTTGAGGCAGGCTGCCTGGGCTAGTAGAGCA	motif identification
pmIoTrxL1-76p1	TGTTGTGCAGTTGGTCTGGGCCATGTAAGATGCTAAGCCC	Point-mutation and
pmIoTrxL1-76p2	ATCTTACATGGCCCAGACCAACTGCACAACATCAACAACG	trapping experiment
pmIoTrxL1-79p1	GTTGGTGTGGGCCATCTAAGATGCTAAGCCCAATCCTTGC	Point-mutation and
pmIoTrxL1-79p2	GGGCTTAGCATCTTAGATGGCCCACACCAACTGCACAACA	trapping experiment

Table S1 continued…

**Primer name**	**Oligonucleotide sequences**	**Experiments**
pmIoTrxL3-38 p1	CTTGGTGTGGACCATCCAAGATGATGGCTCCTACATTCGC	Point-mutation and
pmIoTrxL3-38 p2	GGAGCCATCATCTTGgATGGTCCACACCAAGTGGCGTAGA	trapping experiment
pmIoTrxL3-35p1	CTACGCCACTTGGTCTGGACCATcCAAGATGATGGCTCCT	Point-mutation and
pmIoTrxL3-35p2	ATCTTGgATGGTCCAGACCAAGTGGCGTAGAAGTCAATGA	trapping experiment
pmIoTrxL2-34p1	GTGGTGTGGTCCATCCCGCGTGATTTCGCCAATCTTTGAG	Point-mutation and
pmIoTrxL2-34p2	GCGAAATCACGCGGGATGGACCACACCACGTCGCCCAGAA	trapping experiment
pmIoTrxL2-31p1	TCTGGGCGACGTGGTCTGGTCCATCCCGCGTGATTTCGCC	Point-mutation and
pmIoTrxL2-31p2	ACGCGGGATGGACCAGACCACGTCGCCCAGAAGTCGAATA	trapping experiment
PAL-1F	CCGCTGGTTCCGCGTGGATCCATGGTTGCAAAGTCAGACGGTC	*S*-nitrosylation
PAL-1R	CTCGAGTGCGGCCGCAAGCTTTCCTTCACGCTATCCATTCTCC	
PAL_2 F	CCGCTGGTTCCGCGTGGATCCATGGTAGTCAACTCAGACGGC	*S*-nitrosylation
PAL_2 R	CTCGAGTGCGGCCGCAAGCTTTTCCCACCGTCCAGTCATTC	
4-CL- F	CCGCTGGTTCCGCGTGGATCCATGGCCGTGGGACTGGTCCAAC	*S*-nitrosylation
4-CL- R	CTCGAGTGCGGCCGCAAGCTTGTTGCACCCATGCAAAGAGATA	
sps-1 F	CCGCTGGTTCCGCGTCGTGGATCCATGGTTGCTGCTCCAGGTT	*S*-nitrosylation
sps-1R	CTCGAGTGCGGCCGCAAGCTTGGAGAGTGGAAAACGGGGTA	
IoTrxR- F	CCGCTGGTTCCGCGTGGATCCATGGCACCGATCAATGGTACGG	denitrosylation assay
IoTrxR-R	CTCGAGTGCGGCCGCAAGCTTGTGAGTGGGGTGTGAGAGAG	
IoTrx-L1 F	CCGCTGGTTCCGCGTGGATCCATGACGGTCACCGTAGTCAAGA	denitrosylation assay
IoTrx- L1 R	CTCGAGTGCGGCCGCAAGCTTAGACTCGGTGGCCATCTTGCGT	
IoTrx-L2 F	CCGCTGGTTCCGCGTGGATCCATGGCCAATAACTTAAACGAAAT	denitrosylation assay
IoTrx-L2 R	CTCGAGTGCGGCCGCAAGCTTATTGGTGCCGTAACTCACAC	
IoTrx-L3 F	CCGCTGGTTCCGCGTGGATCCATGACGGTCACCGTAGTCAAG	denitrosylation assay
IoTrx-L3 R	CTCGAGTGCGGCCGCAAGCTTTACACCCAATCTCCTCCCGT	
GSNOR F	CCG CTG GTT CCG CGTGGATCCATGTCAGAAACTGCAG GA AAAATCATCAAGTG	Cloning and expression
GSNOR R	CTCGAGTGCGGCCGCAAGCTTTTAGAGTTCGCTCATATCAACAAC GCAACG	
pmGSNOR 1	ATGGTACATCTCGGTTCACGTCCAAGGGC	Site-directed mutagenesis
pmGSNOR2	GTGTAGGGACTGGCCCTTGGACGTGAACC	of GSNOR
baitIoTrxL1F	CATGGAGGCCGAATTCATGTCAGGCCTTCGCTTCGTACC	yeast two hybrid assay
baitIoTrxL1R	GCAGGTCGACGGATCCTCATAGCCAATTCACTTTCTCTGCCAACG	
baitIoTrxL3 F	CATGGAGGCCGAATTCATGGCCAATAACTTAAACGAAATATC	yeast two hybrid assay
baitIoTrxL3R	GCAGGTCGACGGATCCATTGGTGCCGTAACTCACAC	
preyGSNOR F	TGGCCATTATGGCCCGGGATGTCAGAAACTGCAGGAAAAATCA	yeast two hybrid assay
preyGSNOR R	GACATGTTTTTTCCCGGGTTAGAGTTCGCTCATATCAACA	
